# Alterations of Bio-elements, Oxidative, and Inflammatory Status in the Zinc Deficiency Model in Rats

**DOI:** 10.1007/s12640-015-9571-7

**Published:** 2015-11-18

**Authors:** Urszula Doboszewska, Bernadeta Szewczyk, Magdalena Sowa-Kućma, Karolina Noworyta-Sokołowska, Paulina Misztak, Joanna Gołębiowska, Katarzyna Młyniec, Beata Ostachowicz, Mirosław Krośniak, Agnieszka Wojtanowska-Krośniak, Krystyna Gołembiowska, Marek Lankosz, Wojciech Piekoszewski, Gabriel Nowak

**Affiliations:** Institute of Pharmacology, Polish Academy of Sciences, Smętna 12, 31-343 Kraków, Poland; Faculty of Pharmacy, Jagiellonian University Medical College, Medyczna 9, 30-688 Kraków, Poland; Faculty of Physics and Applied Computer Sciences, AGH University of Science and Technology, Mickiewicza 30, 30-059 Kraków, Poland; Faculty of Chemistry, Jagiellonian University, Ingardena 3, 30-060 Kraków, Poland

**Keywords:** Zinc deficiency, Zinc, Iron, Oxidation, Inflammation, Glutamate

## Abstract

Our previous study showed that dietary zinc restriction induces depression-like behavior with concomitant up-regulation of the *N*-methyl-d-aspartate receptor (NMDAR). Because metal ions, oxidative stress, and inflammation are involved in depression/NMDAR function, in the present study, bio-elements (zinc, copper, iron, magnesium, and calcium), oxidative (thiobarbituric acid-reactive substances; protein carbonyl content), and inflammatory (IL-1α, IL-1β) factors were measured in serum, hippocampus (Hp), and prefrontal cortex (PFC) of male Sprague–Dawley rats subjected to a zinc-adequate (ZnA) (50 mg Zn/kg) or a zinc-deficient (ZnD) (3 mg Zn/kg) diet for 4 or 6 weeks. Both periods of dietary zinc restriction reduced serum zinc and increased serum iron levels. At 4 weeks, lowered zinc level in the PFC and Hp as well as lowered iron level in the PFC of the ZnD rats was observed. At 6 weeks, however, iron level was increased in the PFC of these rats. Although at 6 weeks zinc level in the PFC did not differ between the ZnA and ZnD rats, extracellular zinc concentration after 100 mM KCl stimulation was reduced in the PFC of the ZnD rats and was accompanied by increased extracellular iron and glutamate levels (as measured by the in vivo microdialysis). The examined oxidative and inflammatory parameters were generally enhanced in the tissue of the ZnD animals. The obtained data suggest dynamic redistribution of bio-elements and enhancement of oxidative/inflammatory parameters after dietary zinc restriction, which may have a link with depression-like behavior/NMDAR function/neurodegeneration.

## Introduction

Zinc is the second (after iron) most prevalent trace element in the human body. Its importance has been demonstrated in many physiological processes. Zinc is crucial for normal development and function of cell-mediated immunity. Zinc deficiency primarily has an impact on T cells function and affects Th1 and (to a lesser extend) Th2 cytokines production (Bonaventura et al. 2015). Zinc deficiency influences also production of interleukin (IL)-1β by macrophages (Bonaventura et al. 2015). IL-1β plays a key role in the damaging inflammatory response in a variety of diseases (Dinarello et al. 2012). Altered production of cytokines during zinc deficiency may lead to inflammation, e.g., zinc depletion from macrophages induces IL-1β secretion and activates NLRP3 inflammasome (Summersgill et al. 2014). Moreover, zinc which is (in contrast to iron) redox inactive metal, serves as an important component of antioxidant defense. It contributes to maintaining redox balance through different mechanisms, e.g., is an inhibitor of nicotinamide adenine dinucleotide phosphate (NADPH) oxidase, a co-factor of superoxide dismutase (SOD), and induces the generation of cysteine-rich metallothionein, which acts as scavenger of oxidants (Oteiza 2012; Prasad 2014). In contrast, zinc deficiency is associated with increased oxidative stress markers (Prasad 2014).

There is evidence for the involvement of inflammation and oxidative stress in depression (Leonard and Maes 2012; Maes et al. 2012a; Moylan et al. 2014). Depressed patients exhibit increased levels of pro-inflammatory cytokines, e.g., IL-1β, whereas administration of these cytokines (including IL-1β) induces depression-like behavior in experimental animals (Maes et al. 2012a). Both preclinical and clinical data indicate also that depression is accompanied by increased lipid, protein, and DNA peroxidation (Siwek et al. 2013a). Oxidative stress and inflammation may contribute to depression through effects on the glutamatergic system (Marsden 2011). Pro-inflammatory cytokines, such as IL-1β, and reactive oxygen species can enhance the activity of indoleamine 2,3-dioxygenase (IDO), an enzyme which catabolizes tryptophan, the primary precursor of serotonin, into kynurenine, which is further broken down into kynurenic acid and quinolinic acid. While kynurenic acid is the ionotropic glutamate *N*-methyl-d-aspartate receptor (NMDAR) endogenous antagonist, quinolinic acid is a strong agonist of NMDAR (Leonard and Maes 2012; Maes et al. 2012a; Felger and Lotrich 2013; Myint and Kim 2014). Therefore, inflammation and oxidative stress via activation of IDO pathway may lead to abnormal regulation of glutamate transmission through NMDAR, a phenomenon implicated in the pathophysiology of depression (Sanacora et al. 2012; Ghasemi et al. 2014). Moreover, a mixture of pro-inflammatory cytokines, containing IL-1β, was shown to increase glutamate release (Ida et al. 2008). This observation provides another route by which cytokines may influence the glutamatergic system and conceivably lead to depression.

In addition to oxidative/inflammatory status, NMDAR is a subject to modulation by a number of agents including metal ions (Szewczyk et al. 2012). At resting membrane potential magnesium blocks NMDAR channel, prohibiting calcium influx. Depletion of magnesium block during depolarization allows calcium to enter the postsynaptic neuron. Under certain conditions, magnesium and calcium can block/permeate the channel (Mayer and Westbrook 1987). Zinc is a potent inhibitor of NMDAR (Paoletti et al. 2009). Also copper and iron were found to inhibit this receptor (Vlachova et al. 1996; Nakamichi et al. 2002; Stys et al. 2012). Of note, relationships between altered homeostasis of bio-elements as well as increased oxidative/inflammatory status and NMDAR function were implicated in depressive disorders (Marsden 2011; Leonard and Maes 2012; Serefko et al. 2013; Mlyniec et al. 2014a, 2015).

Recently, a low dietary zinc intake emerged as a risk factor for depression (Vashum et al. 2014). Zinc deficiency is regarded as a public health problem (Jurowski et al. 2014). It induces psychopathological symptoms that correspond to depression symptoms (Szewczyk 2013; Hagmeyer et al. 2015). We have previously shown that depression-like behavior induced by dietary zinc restriction in rats is associated with up-regulation of NMDAR in brain regions (hippocampus, Hp and prefrontal cortex, PFC) (Doboszewska et al. 2015). Because alterations of metals as well as oxidative/inflammatory status may be linked to abnormal NMDAR function and depression, here we measured bio-elements (zinc, copper, calcium, magnesium, and iron) and oxidation/inflammation parameters (thiobarbituric acid-reactive substances (TBARS), protein carbonyl content (PCC), IL-1α and IL-1β) in serum, the Hp and PFC of rats following a zinc-deficient (ZnD) diet administration. Moreover, extracellular zinc, iron, and glutamate levels were measured.

## Materials and Methods

### Animals and Diet

All procedures were conducted according to the National Institutes of Health Animal Care and Use Committee guidelines and were approved by the Ethics Committee of the Institute of Pharmacology, Krakow. In the study, 40 male Sprague–Dawley rats (Charles River Laboratories, Erkrath, Germany) were used that were delivered at the age of 4 weeks. Upon arrival, the rats were habituated to the laboratory conditions for 1 week. During the habituation phase, the rats were fed a standard diet with 35 mg Zn/kg. Following the habituation phase, the animals were divided into 4 groups; each group consisted of *n* = 10 rats that were fed a zinc-adequate (ZnA) diet of 50 mg Zn/kg or a ZnD diet of 3 mg Zn/kg, for 4 or 6 weeks. Detailed specification of elements: zinc, copper, calcium, magnesium and iron for the ZnA and ZnD diets is provided in Table 1. The diets were purchased from Altromin GmbH (Lage, Germany). The animals were housed 5 per cage in a controlled environment (temperature 22 ± 2 °C, 12 h light/dark cycle, 40–50 % humidity) with free access to food and water. The body weight of each rat was measured weekly. Additional group (*n* = 9) of a 4-week male Sprague–Dawley rats (Charles River Laboratories) was used in the in vivo microdialysis study. Before the in vivo microdialysis, the rats were applied a 6-week diet regimen. Briefly, following a 1-week habituation period, the rats were divided into groups (*n* = 4–5) that received a ZnA or a ZnD diet for 6 weeks.

**Table 1:** The amounts of bio-elements: zinc (Zn), copper (Cu), calcium (Ca), magnesium (Mg), and iron (Fe) in the zinc-adequate (ZnA) and zinc-deficient (ZnD) diets

	Units	ZnA diet	ZnD diet
Zn	mg/kg	50	3
Cu	mg/kg	5	5
Ca	mg/kg	9482	9513
Mg	mg/kg	709	716
Fe	mg/kg	179	179

### Tissue Processing

Following 4 or 6 weeks of the ZnA or ZnD diet, the rats were killed by rapid decapitation; their brains were rapidly dissected and immersed in cooled (2–8 °C) 0.9 % sodium chloride (0.9 % NaCl) solution. Complete hippocampus (Hp) (dorsal and ventral) and prefrontal cortex (PFC) were dissected on a cold plate, immediately frozen on dry ice and stored at −80 °C until further analysis. The trunk blood was collected into tubes without anti-coagulant. The blood was allowed to clot for 15–20 min and was centrifuged for 30 min at 1800 rpm at 4 °C. The resulting supernatant (serum) was quickly pipetted into tubes that were stored at −80 °C until analysis.

### Total Reflection X-Ray Fluorescence

Zinc, copper, and iron concentrations were measured in serum, the Hp, and PFC using total reflection X-ray fluorescence (TXRF) method, as described previously (Opoka et al. 2010). The Hp and PFC were weighted and digested in 100–300 μL (depending on the mass of the sample) of the concentrated nitric acid. As an internal standard, selenium standard was added, so that the final concentration was 5 mg/l for serum and 30–50 mg/kg for brain tissues. From the resulting solutions, 5 μl was pipetted on reflectors made of clean glass used for TXRF analysis. Concentrated nitric acid, suprapur quality, additionally cleaned by subboiling distillation procedure, was purchased from Merck (Darmstadt, Germany). Selenium standard, 1000 mg/l selenium in nitric acid, was purchased from Sigma-Aldrich (Saint Louis, Missouri, United States). NANOHUNTER TXRF Spectrometer from Rigaku (Japan) was used. Mo X-ray tube (50 kV, 0.8 mA) was applied. The results are presented in mg/l (for serum) or mg/kg wet weight of brain tissue.

### Flame Atomic Absorption Spectroscopy

Calcium and magnesium concentrations were measured in serum, the Hp, and PFC using flame atomic absorption spectroscopy, as published previously (Kopanski et al. 2000). The Hp and PFC were weighted (0.018–0.198 g) and digested in 1.5 ml of concentrated nitric acid (SupraPur—Merck). Brain tissue samples were mineralized using water bath for 5 h at 80 °C. After cooling the samples, demineralized water was added to the total volume of 5 ml. Magnesium was measured at wavelength of 285.2 nm and calcium at 422.7 nm, both with deuterium (D2) background correction. The results are presented in mg/l (for serum) or mg/kg wet weight of brain tissue.

### Endocrine, Oxidative Stress, and Inflammation Assays

Serum corticosterone (CORT), oxidative, and inflammatory parameters were measured using commercially available kits, according to the manufacturer’s protocols. Serum CORT concentration was determined by enzyme-linked immunoassay (ELISA) using Corticosterone Rat/Mouse Elisa Kit (Demeditec Diagnostics GmbH, Kiel, Germany). The levels of lipid and protein peroxidation were measured in serum, the Hp, and PFC using thiobarbituric acid-reactive substances (TBARS) Assay kit or Protein Carbonyl Colorimetric Assay Kit (Cayman Chemical, Ann Arbor, MI, USA), respectively. To determine lipid peroxidation, the malondialdehyde (MDA)-thiobarbituric acid (TBA) adduct was measured fluorometrically at an excitation wavelength of 530 nm and an emission wavelength of 550 nm. The levels of IL-1α and IL-1β were measured in serum, the Hp, and PFC using Rat IL-1α or Il-1β ELISA kit (RayBiotech, Norcross, GA, USA).

### In Vivo Microdialysis

Following 6 weeks of the ZnA or ZnD diet, in vivo microdialysis was performed. Five days before the microdialysis, the rats were anesthetized with chloral hydrate (400 mg/kg) and individually placed in a stereotaxic apparatus (David Kopf Instruments, Tujunga, CA, USA). Their skull was exposed, a burr hole was drilled, and a guide cannula (AgnTho’s, Sweden) was implanted in the rat PFC with coordinates: AP  = + 2.8; *L* = + 0.8; *V* = −2.4, based on the brain atlas (Paxinos and Watson 1998). The guide cannula was secured with dental cement (Dentalon; Heraeus-Kulzer GmbH, Germany) and a screw. After the surgical operation, each rat was housed individually. Five days after implantation of the guide cannula, a microdialysis probe (3 mm, AgnTho’s, Sweden) was inserted into the PFC through the guide cannula. The microdialysis probe was connected with the microdialysis pump, which infused an artificial cerebrospinal fluid (aCSF) containing 147 mM NaCl, 4.0 mM KCl, 1.0 mM MgCl_2_, 2.2 CaCl_2_, and pH = 7.4 with a flow of 2 μl/min. After a 2 h washout period, the fractions were collected for 2 h (to determine the basal extracellular concentrations of zinc, iron, and glutamate). Then, the PFC was perfused with 100 mM KCl in Ringer’s solution for 40 min, and the fractions were collected every 20 min (to determine the extracellular concentrations of zinc, iron, and glutamate after depolarization).

### Analytical Procedures: Microdialysates

The extracellular concentrations of zinc and iron were measured by TXRF method using selenium standard, as described above. The extracellular concentration of glutamate was measured after derivatization with OPA/sulfite reagent. OPA/sulfite glutamate derivative was analyzed using high-performance liquid chromatography (HPLC) method with electrochemical detection. Chromatography was performed using Dionex P580 pump (USA), an LC 4B amperometric detector with a cross-flow detector cell (BAS, IN, USA), and a HR-80 column (4 × 80 mm, 3 µm; ESA, USA). The mobile phase consisted of 0.1 M phosphate buffer with pH 3.6 and 5 % methanol. The flow rate was 0.8 ml/min, and the applied potential of 3-mm glassy carbon electrode was +650 mV at a sensitivity of 5 nA/V. Glutamate derivative concentration was calculated by comparing its peak area with respective standard and was processed by Chromax 2005 (Pol-Lab, Warsaw, Poland) software run on a personal computer.

### Statistics

Data were analyzed using unpaired, two-tailed Student’s *t* test or Repeated Measures ANOVA followed by a Bonferroni post hoc test. All results are presented as the mean ± SEM. *P* < 0.05 was considered statistically significant with 95 % confidence (Prism ver. 4, GraphPad Software, San Diego, CA, USA).

## Results

### The Effects of Dietary Zinc Restriction on Body Weight

**Fig. 1:**
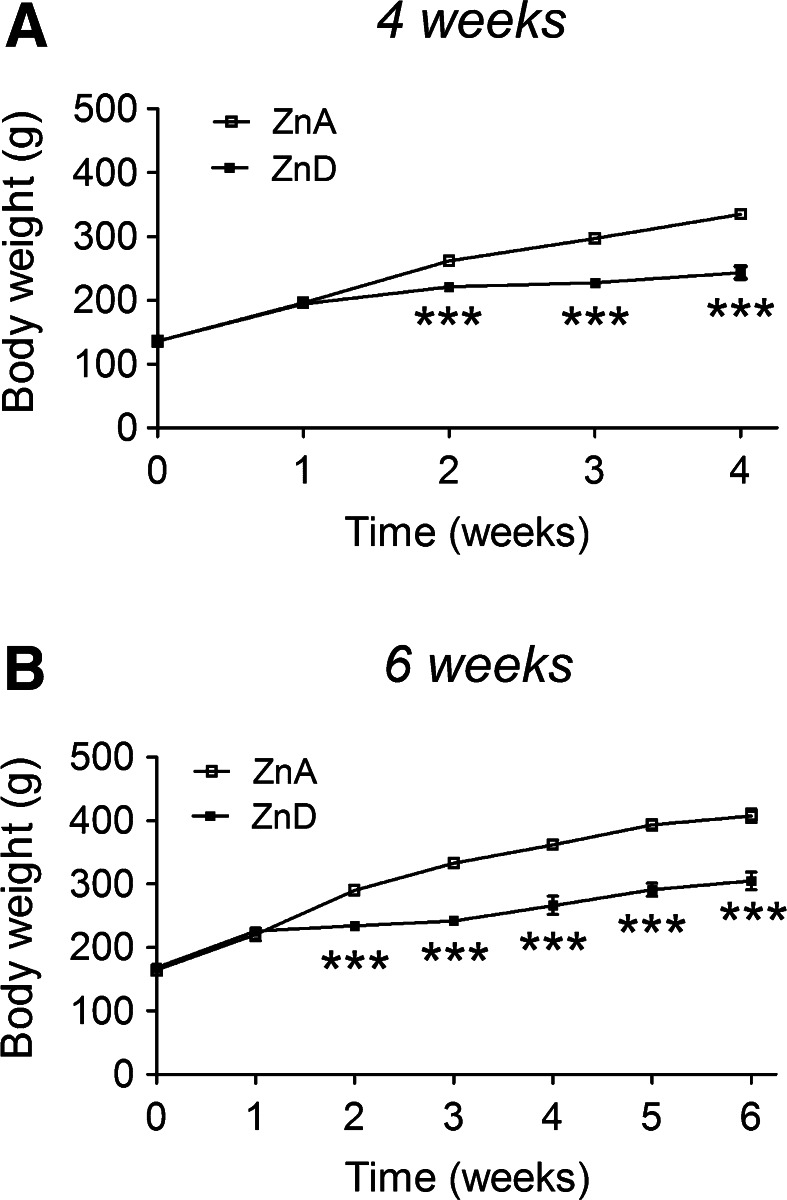
The effects of a 4-week **a** or a 6-week, **b** dietary zinc restriction on body weight. Rats were fed a zinc-adequate (ZnA) diet (50 mg Zn/kg) or a zinc-deficient (ZnD) diet (3 mg Zn/kg) for 4 or 6 weeks. Values are expressed as the mean ± SEM, *n* = 10 per group. Repeated measures ANOVA demonstrated a significant interaction [*F*(4,72) = 52.51, *p* < 0.0001], a significant effect of the diet [*F*(1,18) = 58.72, *p* < 0.0001] and a significant effect of time [*F*(4,72) = 464.16, *p* < 0.0001] on body weight following a 4-week ZnD diet. Repeated measures ANOVA demonstrated a significant interaction [*F*(6,108) = 31.09, *p* < 0.0001], a significant effect of the diet [*F*(1,108) = 49.41, *p* < 0.0001], and a significant effect of time [*F*(6,108) = 240.09, *p* < 0.0001] on body weight following a 6-week ZnD diet. ***p < 0.001 versus respective ZnA (Bonferroni post hoc test)

During the zinc regimen, the rats displayed a typical early sign of zinc deficiency of growth retardation (Prasad 2012). A gradual increase in body weight in both groups in the course of time was observed, but the body weight was significantly reduced in rats fed the ZnD diet for 4 weeks, compared to rats fed the ZnA diet, in the second, third, and fourth weeks (Fig. 1a). The body weight was also significantly reduced in rats fed the ZnD diet for 6 weeks. This effect emerged in the second week and persisted to the sixth week (Fig. 1b).

### The Effects of a 4-week Dietary Zinc Restriction on Zn, Cu, Ca, Mg, Fe, CORT, TBARS, and PCC Levels in Serum

**Fig. 2:**
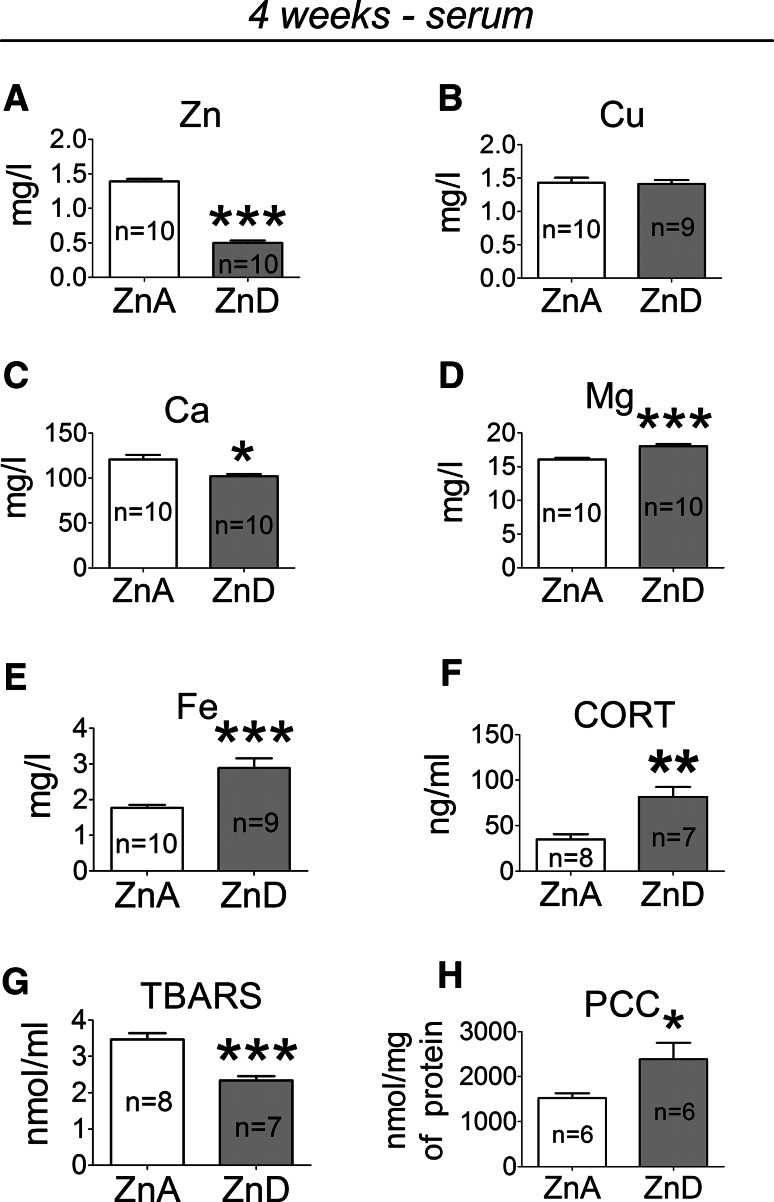
The effects of a 4-week dietary zinc restriction on zinc (Zn) (**a**), copper (Cu) (**b**), calcium (Ca) (**c**), magnesium (Mg) (**d**), iron (Fe) (**e**), corticosterone (CORT) (**f**), thiobarbituric acid-reactive substances (TBARS) (**g**) and protein carbonyl content (PCC) (**h**) concentrations in serum. Values are expressed as the mean ± SEM. Data were analyzed by Student’s *t* test. *p < 0.05, **p < 0.01, ***p < 0.001 versus respective ZnA. Statistical details: Zn [*p* < 0.0001, *t*(18) = 17.64], Cu [*p* = 0.8373, *t*(17) = 0.2085], Ca [*p* = 0.0037, *t*(18) = 3.329], Mg [*p* = 0.0002, *t*(18) = 4.703], Fe [*p* = 0.0007, *t*(17) = 4.112], CORT [*t*(13) = 3.924, *p* = 0.0017], TBARS [*t*(13) = 5.260, *p* = 0.0002], PCC [*t*(10) = 2.256, *p* = 0.0477]

A 4-week ZnD diet significantly decreased serum zinc (Fig. 2a) and calcium (Fig. 2c) concentrations, increased magnesium (Fig. 2d) and iron (Fig. 2e) concentrations but did not affect serum copper concentration (Fig. 2b). Moreover, a 4-ZnD diet significantly increased serum CORT (Fig. 2f) and PCC (Fig. 2h) concentrations but decreased TBARS concentration (Fig. 2g).

### The Effects of a 4-week Dietary Zinc Restriction on Zn, Cu, Ca, Mg, Fe, TBARS, PCC, IL-1α, and IL-1β Levels in the Hp and PFC

**Fig. 3:**
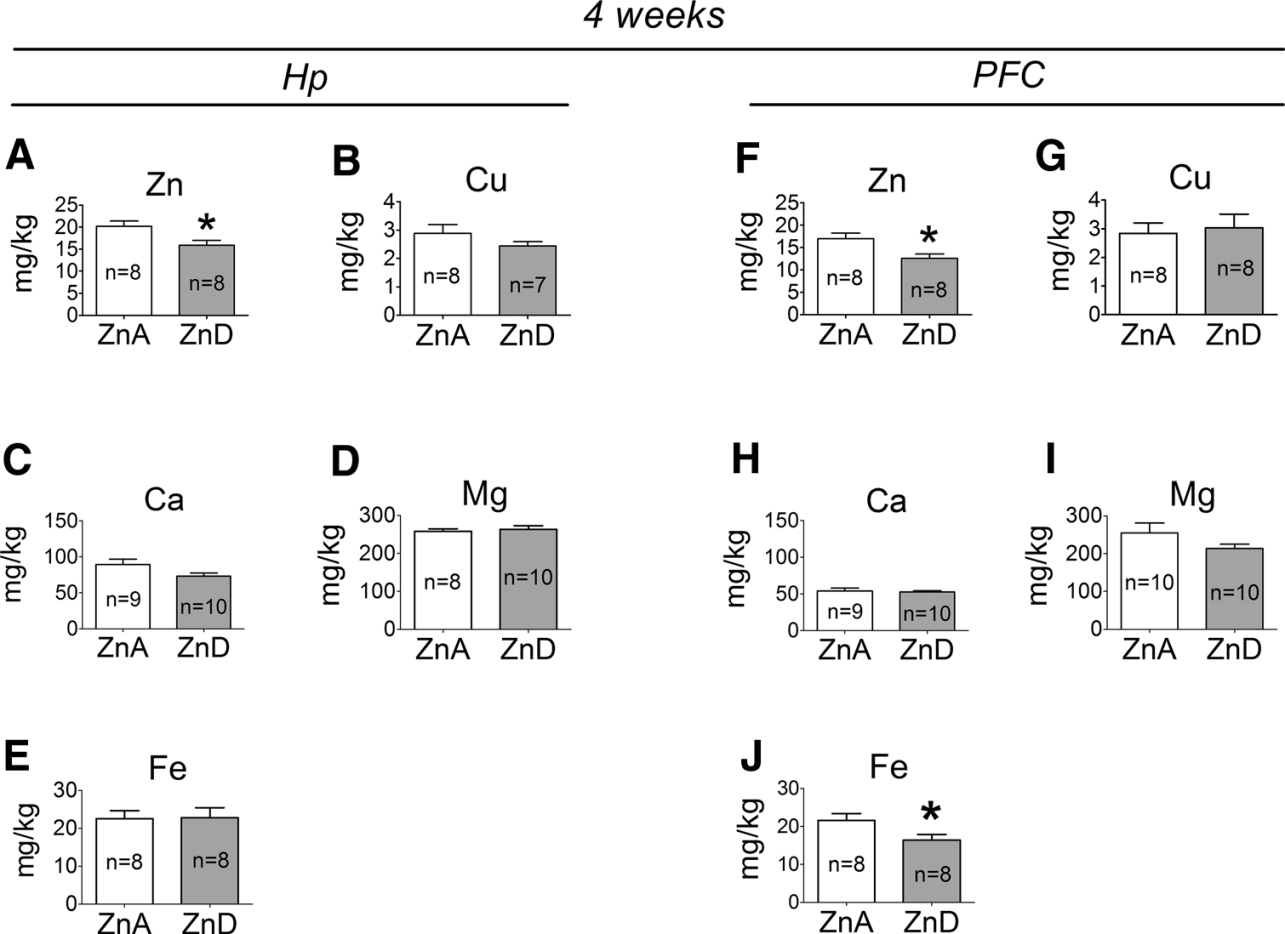
The effects of a 4-week dietary zinc restriction on Zn (**a**), Cu (**b**), Ca (**c**), Mg (D), Fe (**e**) levels in the hippocampus (Hp) and on Zn (**f**), Cu (**g**), Ca (**h**), Mg (**i**), Fe (**j**) levels in the prefrontal cortex (PFC). Values are expressed as the mean ± SEM. Data were analyzed by Student’s *t* test. *p < 0.05 versus respective ZnA. Statistical details: *Hp*: Zn [*p* = 0.0199, *t*(14) = 2.628], Cu [*p* = 0.2402, *t*(13) = 1.232], Ca [*p* = 0.0777, *t*(17) = 1.877], Mg [*p* = 0.6797, *t*(16) = 0.4206], Fe [*p* = 0.9477, *t*(14) = 0.06674]. *PFC*: Zn [*p* = 0.0154, *t*(14) = 2.758], Cu [*p* = 0.7520, *t*(14) = 0.3223], Ca [*p* = 0.7024, *t*(17) = 0.3886], Mg [*p* = 0.1787, *t*(18) = 1.399], Fe [*p* = 0.0397, *t*(14) = 2.268]

**Fig. 4:**
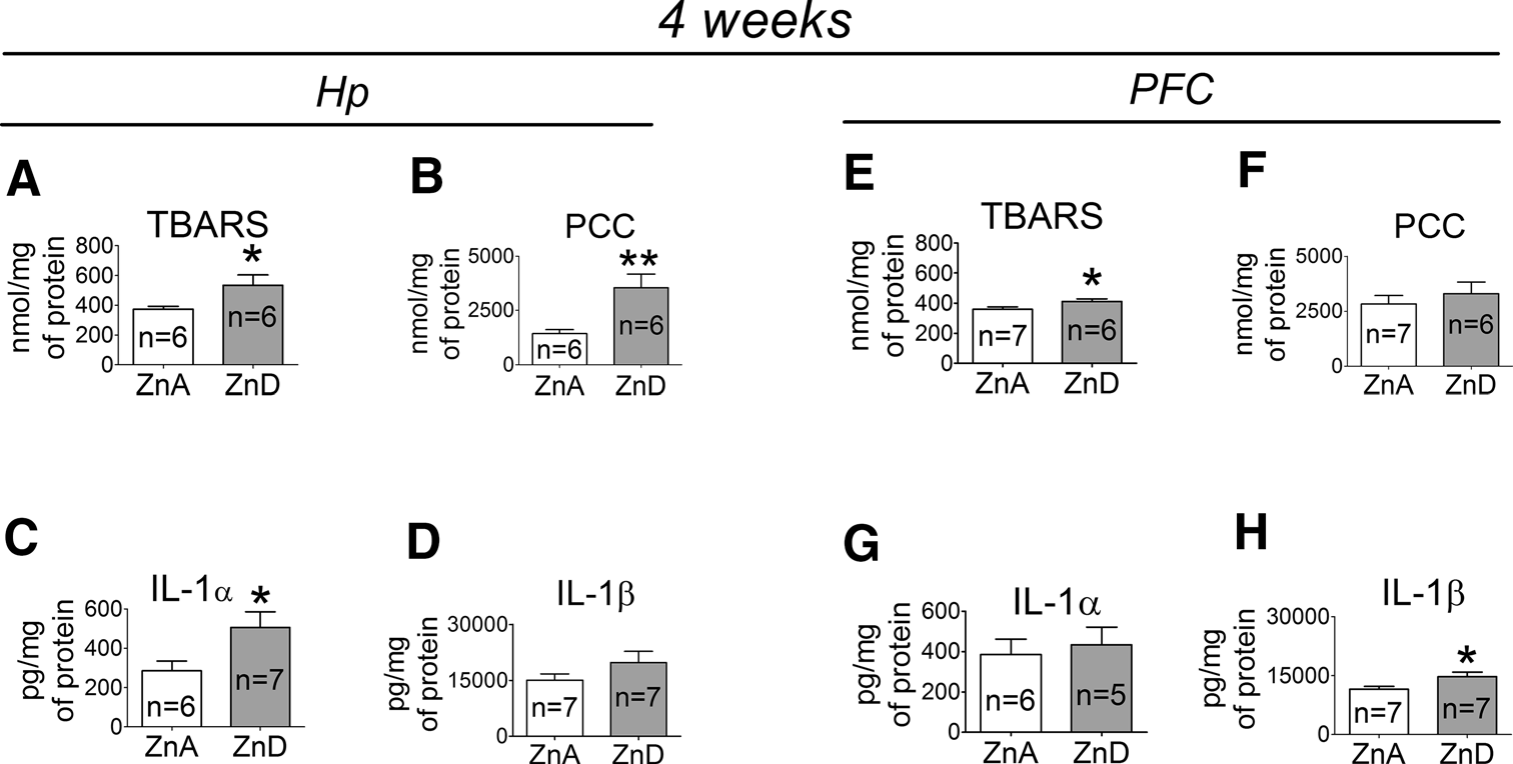
The effects of a 4-week dietary zinc restriction on TBARS (**a**), PCC (**b**), interleukin-1 alpha *(*IL-1α) (**c**), interleukin-1 beta (Il-1β) (**d**) levels in the Hp and on TBARS (**e**), PCC (**f**), IL-1α (**g**), Il-1β (**h**) levels in the PFC. Values are expressed as the mean ± SEM. Data were analyzed by Student’s *t* test. *p < 0.05, **p < 0.01 versus respective ZnA. Statistical details: *Hp*: TBARS [*p* = 0.0499, *t*(10) = 2.230], PCC [*p* = 0.0096, *t*(10) = 3.192], IL-1α [*p* = 0.0438, *t*(11) = 2.277], IL-1β [*p* = 0.1842, *t*(12) = 1.409]. *PFC*: TBARS [*p* = 0.0443, *t*(11) = 2.271], PCC [*p* = 0.4878, *t*(11) = 0.7179], IL-1α [*p* = 0.6887, *t*(9) = 0.4138], IL-1β [*p* = 0.0389, *t*(12) = 2.318]

A 4-week ZnD diet significantly decreased zinc levels in the Hp (Fig. 3a) and PFC (Fig. 3f) of rats. In addition, decreased iron level was observed in the PFC (Fig. 3j), but not in the Hp (Fig. 3e) of the ZnD rats. At 4 weeks, the levels of copper did not significantly differ in the Hp (Fig. 3b) or PFC (Fig. 3g) between the ZnD and control animals. Similarly, the concentrations of calcium (Fig. 3c, h) or magnesium (Fig. 3d, i) in the Hp or PFC did not significantly differ between the ZnA and ZnD groups. TBARS (Fig. 4a), PCC (Fig. 4b), and IL-1α (Fig. 4c) but not IL-1β (Fig. 4d) levels were significantly increased in the Hp of the ZnD rats at 4 weeks. The levels of TBARS (Fig. 4e) and IL-1β (Fig. 4h) were also significantly increased in the PFC of the ZnD group, but the levels of PCC (Fig. 4f) or IL-1α (Fig. 4g) did not significantly differ in the PFC between the ZnD and ZnA animals at 4 weeks.

### The Effects of a 6-week Dietary Zinc Restriction on Zn, Cu, Ca, Mg, Fe, CORT, and TBARS Levels in Serum

**Fig. 5:**
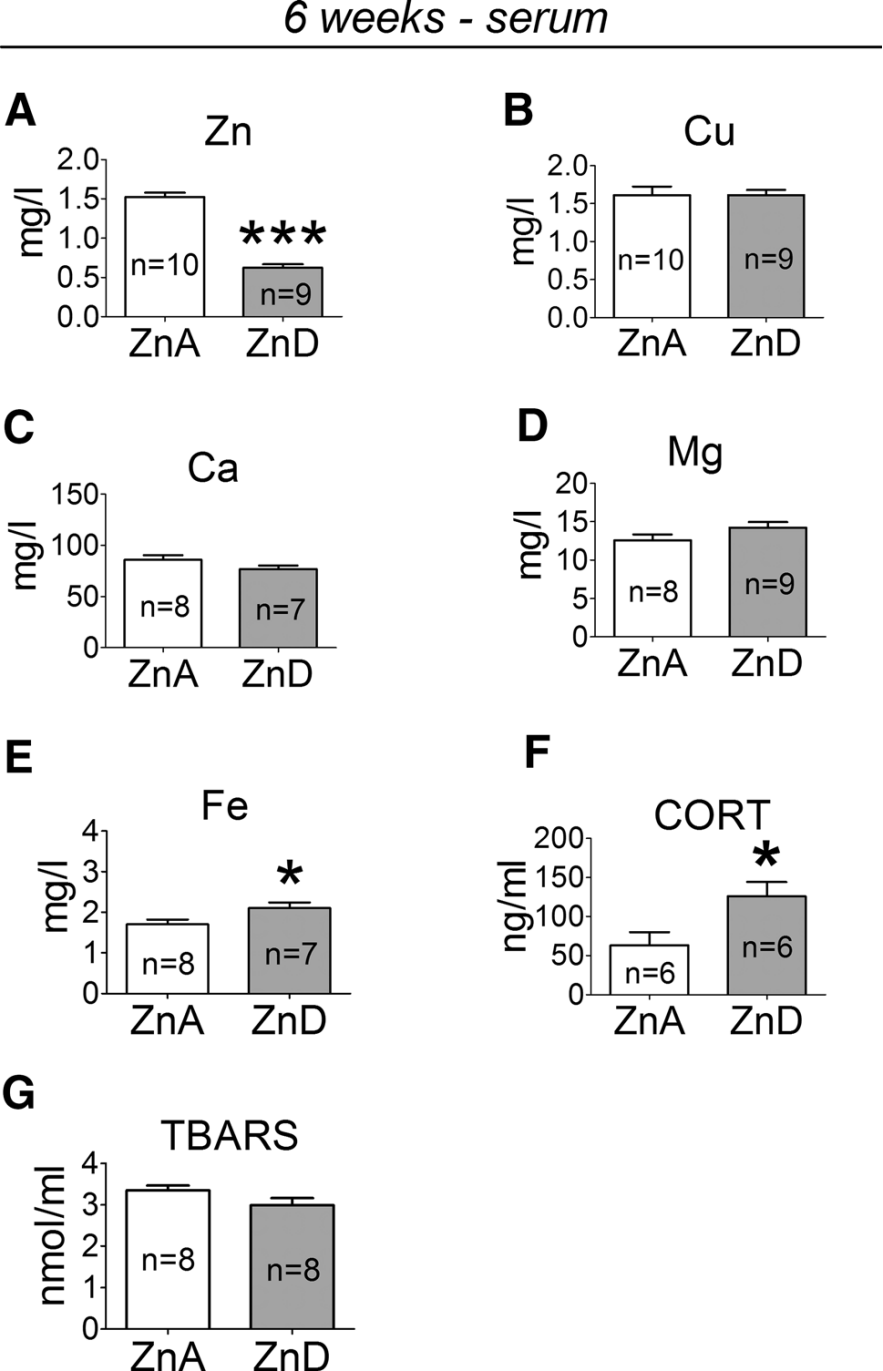
The effects of a 6-week dietary zinc restriction on Zn (**a**), Cu (**b**), Ca (**c**), Mg (**d**), Fe (**e**), CORT (**f**), and TBARS (**g**) concentrations in serum. Values are expressed as the mean ± SEM. Data were analyzed by Student’s *t* test. *p < 0.05, ***p < 0.001 versus respective ZnA. Statistical details: Zn [*p* < 0.0001, *t*(17) = 12.80], Cu [*p* = 0.9929, *t*(17) = 0.009016], Ca [*p* = 0.1231, *t*(13) = 1.649], Mg [*p* = 0.1452, *t*(15) = 1.537], Fe [*p* = 0.0481, *t*(13) = 2.181], CORT [*t*(10) = 2.490, *p* = 0.0320], TBARS [*t*(14) = 1.661, *p* = 0.1190]

A 6-week ZnD diet significantly decreased serum zinc (Fig. 5a), increased iron (Fig. 5e) and CORT (Fig. 5f) concentrations, but did not significantly affect copper (Fig. 5b), calcium (Fig. 5c), magnesium (Fig. 5d), or TBARS (Fig. 5g) concentrations.

### The Effects of a 6-week Dietary Zinc Restriction on Zn, Fe, and TBARS Levels in the Hp and PFC

**Fig. 6:**
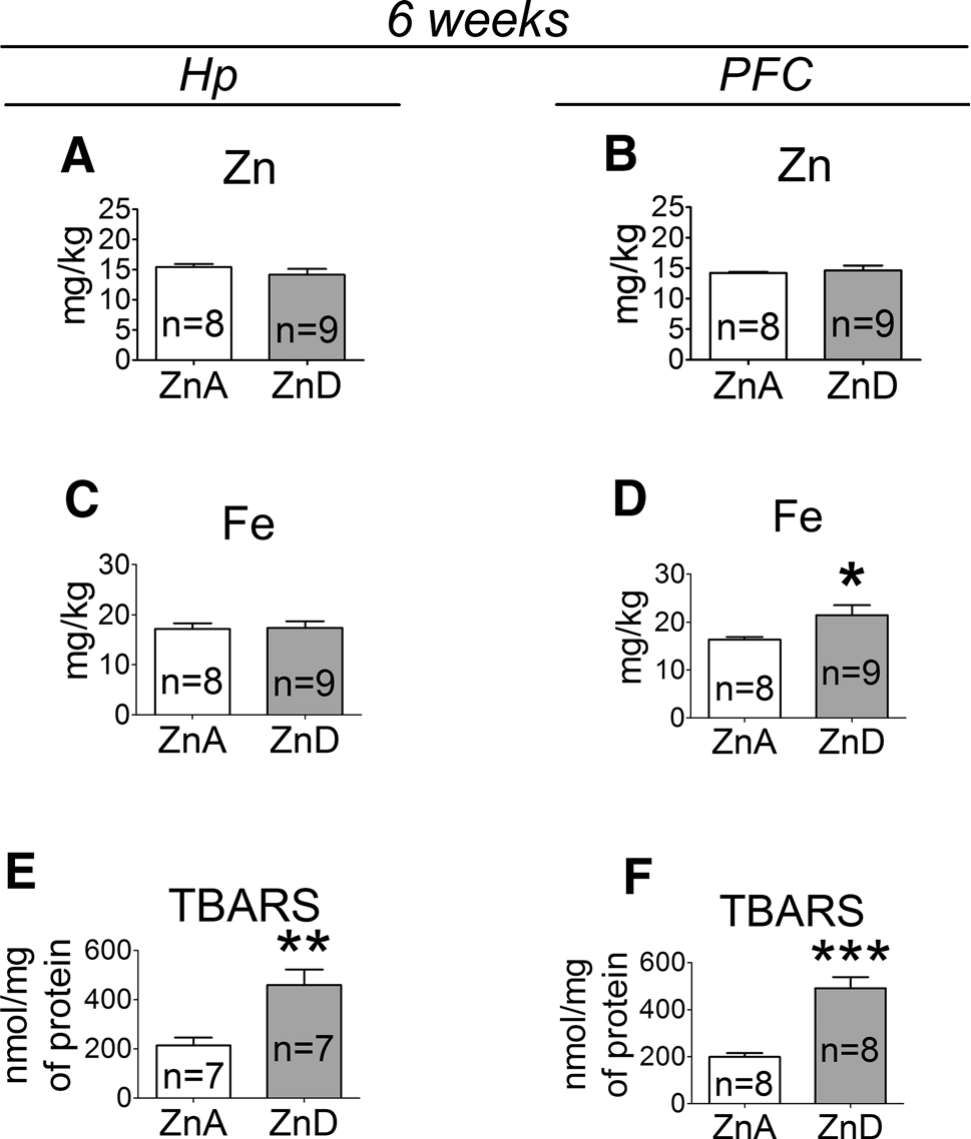
The effects of a 6-week dietary zinc restriction on Zn (**a**), Fe (**c**), TBARS (**e**) levels in the Hp and on Zn (**b**), Fe (**d**) and TBARS (**f**) levels in the PFC. Values are expressed as the mean ± SEM. Data were analyzed by Student’s *t* test. *p < 0.05, **p < 0.01, ***p < 0.001 versus respective ZnA. Statistical details: *Hp*: Zn [*p* = 0.2708, *t*(15) = 1.143], Fe [*p* = 0.9077, *t*(15) = 0.1180], TBARS [*p* = 0.0045, *t*(12) = 3.491]. *PFC*: Zn [p = 0.6603, *t*(15) = 0.4483], Fe [*p* = 0.0429, *t*(15) = 2.212], TBARS [*p* < 0.0001, *t*(14) = 5.751]

At 6 weeks, there were no significant changes in zinc level in the Hp (Fig. 6a) or PFC (Fig. 6b) between the ZnD and control animals, whereas iron level was significantly increased in the PFC (Fig. 6d), but not in the Hp (Fig. 6c) of the ZnD rats. Furthermore, TBARS levels were significantly increased in both the Hp (Fig. 6e) and PFC (Fig. 6f) of the ZnD rats at 6 weeks.

### The Effects of a 6-week Dietary Zinc Restriction on Extracellular Zn, Fe, and Glutamate Levels in the PFC

**Fig. 7:**
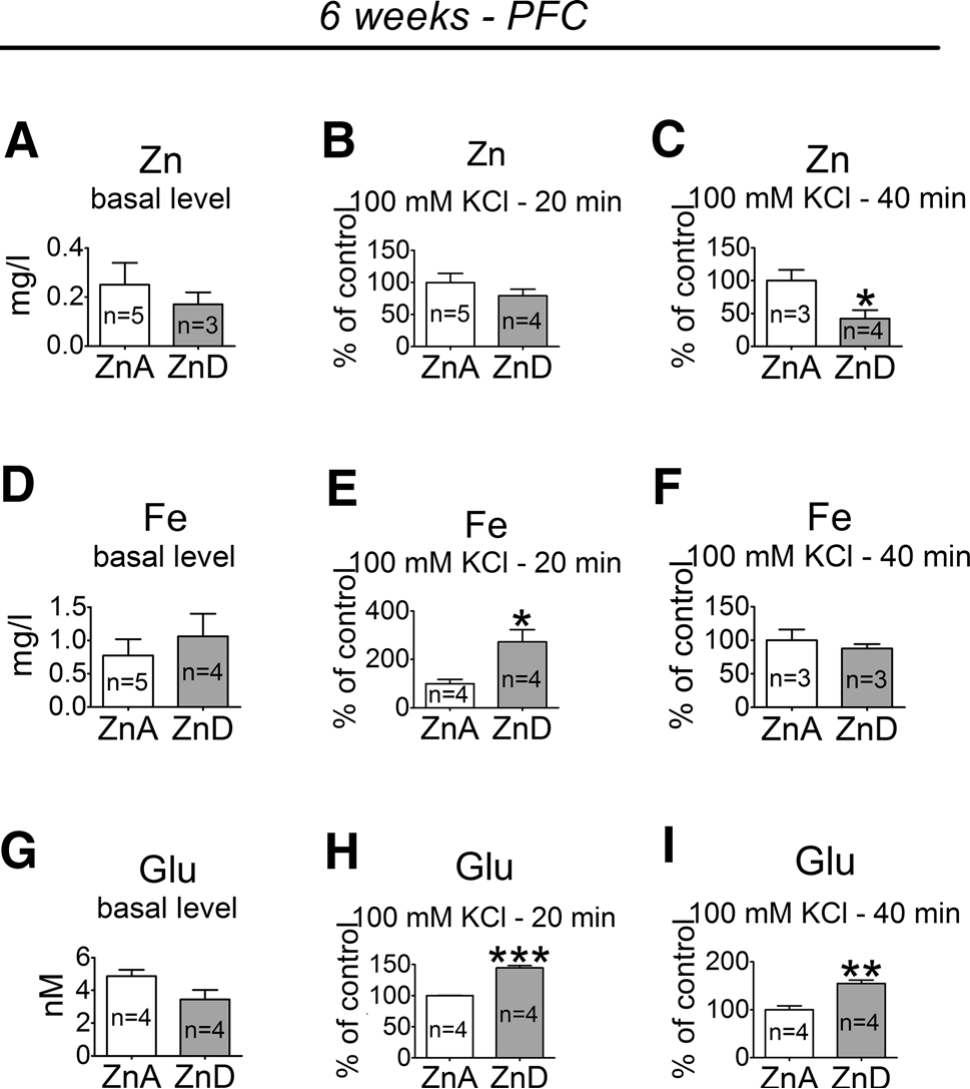
The effects of a 6-week dietary zinc restriction on extracellular Zn, Fe, and glutamate (Glu) concentrations in the PFC: basal extracellular Zn level (**a**), extracellular Zn level after a 20-min stimulation with 100 mM KCl (**b**), extracellular Zn level after a 40-min stimulation with 100 mM KCl (**c**); basal extracellular Fe level (**d**), extracellular Fe level after a 20-min stimulation with 100 mM KCl (**e**), extracellular Fe level after a 40-min stimulation with 100 mM KCl (**f**); basal extracellular Glu level (**g**), extracellular Glu level after a 20-min stimulation with 100 mM KCl (**h**), extracellular Glu level after a 40-min stimulation with 100 mM KCl (**i**). Basal extracellular levels are expressed as the mean ± SEM. The extracellular levels after stimulations (mean ± SEM) are presented as the % of respective ZnA. Data were analyzed by Student’s *t* test. *p < 0.05, **p < 0.01, ***p < 0.001 versus respective ZnA. Statistical details: Zn basal level [*p* = 0.5389, *t*(6) = 0.6515], Zn 20-min stimulation [*p* = 0.2983, *t*(7) = 1.124)], Zn 40-min stimulation [*p* = 0.0386, *t*(5) = 2.786], Fe basal level [*p* = 0.4922, *t*(7) = 0.7125], Fe 20-min stimulation [*p* = 0.0168, *t*(6) = 3.280], Fe 40-min stimulation [*p* = 0.5250, *t*(4) = 0.7036], Glu basal level [*t*(6) = 2.046, *p* = 0.0867], Glu 20-min stimulation [*t*(6) = 13.23, *p* < 0.0001], Glu 40-min stimulation [*t*(6) = 4.901, *p* = 0.0027]

At 6 weeks, the extracellular zinc level in the PFC was significantly decreased in the ZnD group, compared to the ZnA group, after a 40-min stimulation with 100 mM KCl (Fig. 7c). The basal extracellular zinc level (Fig. 7a) or zinc level after a 20-min stimulation with 100 mM KCl (Fig. 7b) did not significantly differ between the ZnD and ZnA groups. The extracellular iron level was significantly increased in the PFC of the ZnD rats after a 20-min (Fig. 7e), but not after a 40-min stimulation (Fig. 7f). The basal extracellular iron level did not differ significantly between the ZnD and ZnA groups (Fig. 7d). Moreover, the basal extracellular glutamate level did not differ significantly between the ZnD and ZnA rats (Fig. 7g). However, after a 20- (Fig. 7h) or a 40-min (Fig. 7i) stimulation, the extracellular level of glutamate was significantly increased in the PFC of the ZnD group, compared to the ZnA group.

## Discussion

We have previously shown that 4 or 6 weeks of the ZnD diet induces depression-like behavior with concomitant alterations within NMDAR signaling pathway (Doboszewska et al. 2015). In the present study, we measured bio-elements and oxidative/inflammatory parameters, which may be associated with depression/NMDAR function, in the ZnD rats.

We found lowered zinc level in the Hp and PFC of rats after a 4-week ZnD diet. Most of the published work demonstrated no changes in total zinc level in brain regions following its chronic restriction (Takeda et al. 2003, 2005, 2008a), but decreased zinc level in the Hp in a response to dietary zinc deficiency has been previously reported (Takeda et al. 2001). Because zinc released from presynaptic terminals of glutamatergic neurons in the cortex and Hp inhibits NMDAR on the postsynaptic side (Paoletti et al. 2009), the decrease in zinc content of these brain regions may have a consequence for NMDAR function and presumably the development of depression. The observation that the time-course of decreased zinc level in brain regions corresponds to the time of occurrence of depression-like behavior and up-regulation of NMDAR subunits (GluN2A, GluN2B) in the Hp of the ZnD rats, which was found in our previous study (Doboszewska et al. 2015) (Table 2), is consistent with this line of thinking.

**Table 2:** Summary of previous findings on the zinc deficiency model in rats

	4 weeks	6 weeks
Behavioral parameters	Increased immobility time in the forced swim test (FST) Anhedonia Reduction of social behavior	Increased immobility time in the FST Anhedonia Reduction of social behavior
Protein levels	*Hp*: ↑ GluN2A, ↑GluN2B, ↓p-CREB, ↓BDNF	*Hp*: ↑ GluN2A, ↑GluN2B, ↓p-CREB, ↓BDNF
	*PFC*: ↔GluN2A, ↔GluN2B, ↔p-CREB, ↔BDNF	*PFC*: ↑ GluN2A, ↔GluN2B, ↓p-CREB, ↓BDNF

Rats were fed a ZnA or a ZnD diet for 4 or 6 weeks. GluN2A, GluN2B—subunits of the glutamate *N*-methyl-d-aspartate receptor; *p*-CREB—phosphorylated cyclic AMP response element-binding protein; BDNF—brain-derived neurotrophic factor; ↑—increased protein level; ↓—decreased protein level; ↔ no effects. Based on Doboszewska et al. 2015

Decreased hippocampal zinc level corresponding to depression-like behavior was found in rats exposed to psychological stress (Dou et al. 2014) and was associated with increased serum CORT concentration (Tao et al. 2013). Increased serum CORT was also demonstrated following zinc restriction (the present data, Watanabe et al. 1992; Chu et al. 2003; Takeda et al. 2007, 2008a, b, Takeda et al. 2012, Mlyniec et al. 2012). Thus, the ZnD diet induces changes in hippocampal zinc and serum CORT similar to those observed after exposure to stress, which is considered as a precipitant of depression.

The lack of differences between zinc level in the Hp or PFC of the ZnD and control rats after prolonged (a 6-week) deprivation suggests dynamic redistribution of the bio-element during its dietary regimen. It should be noted, however, that at 6 weeks of the ZnD diet, a concomitant decrease in evoked zinc release in the PFC was observed, which was accompanied by increased glutamate release (as measured by the in vivo microdialysis). Because we have previously found significantly increased level of NMDAR GluN2A protein in the PFC of rats at 6 weeks of zinc restriction (Doboszewska et al. 2015) (Table 2) (which indicates that a 6-week ZnD diet may alter the function of NMDAR in the PFC), our previous and present observations suggest that zinc deficiency may enhance glutamate signaling through NMDAR.

Reductions in protein expression of zinc transporter 3 (ZnT3) in Brodmann area 9 (which is a part of the frontal cortex) were shown to be significantly associated with elevated depression scores in patients with dementia with Lewy bodies (DLB), Parkinson disease dementia (PDD), and Alzheimer disease (AD) (Whitfield et al. 2015). Because in ZnT3 knockouts, synaptic zinc is undetectable (Cole et al. 1999), these data provide evidence for the role of decreased synaptic zinc in depression. Therefore, decreased evoked zinc release in the PFC of the ZnD rats, suggesting decreased level of synaptic zinc, may be linked to depression-like behavior in the zinc deficiency paradigm.

In the present study, the 4-week ZnD diet reduced iron content of the PFC, whereas an increase in iron content of the PFC was observed after the 6-week ZnD diet. Iron deficiency may lead to abnormal mood and behavior (Kim and Wessling-Resnick. 2014). On the other hand, iron progressively accumulates in the brain with aging (Bilgic et al. 2012; Ramos et al. 2014) and its accumulation in the brain has been implicated in the etiology of numerous neurodegenerative disorders (Schipper 2012; Zheng and Monnot 2012). There is evidence that depression is accompanied by a progressive process of neurodegeneration (Szewczyk et al. 2011; Maes et al. 2012b; Smaga et al. 2014). Magnetic resonance imaging studies in elderly depressed patients have shown that an older age of onset and a greater severity of depression are associated with increased iron deposition in specific brain regions (Steffens et al. 1998). Moreover, exposure to psychological stress was found to increase iron level in the cortex and Hp (Wang et al. 2008; Yu et al. 2011), whereas zinc supplementation was found to decrease iron deposition in the cortex and Hp induced by psychological stress (Li et al. 2012). Thus, the increase in iron level in the brain may be a common effect of prolonged zinc deficiency, psychological stress, and depression.

As mentioned above, we have previously found significantly increased level of NMDAR GluN2A protein in the PFC of rats after the 6-week ZnD diet; however, no differences in the levels of NMDAR GluN2A or GluN2B proteins were observed in the PFC between the ZnD and control animals after the 4-week ZnD diet (Doboszewska et al. 2015) (Table 2). These data, together with the present data concerning increased glutamate release after 6 weeks of the ZnD diet administration, indicate that prolonged (6 weeks) zinc restriction may lead to activation of NMDAR in the PFC. It has been demonstrated that the stimulation of NMDAR induces iron uptake (Cheah et al. 2006). This mechanism may explain iron accumulation in the PFC of rats after 6 weeks, but not after 4 weeks of zinc deprivation. Moreover, at 6 weeks, but not at 4 weeks, we found decreased brain-derived neurotrophic factor (BDNF) protein expression in the PFC of the ZnD rats (Doboszewska et al. 2015) (Table 2). BDNF can ameliorate iron accumulation in neurons (Zhang et al. 2014). Thus, increased iron level in the PFC of rats at 6 weeks might result from decreased level of BDNF.

In addition to decreased zinc release, increased evoked iron release was observed in the PFC of the ZnD rats. Iron transporter ferroportin was found to be expressed in synaptic vesicles (Wu et al. 2004); however, iron homeostasis in the brain has not been clearly defined (Ward et al. 2014). Presumably, the presence of iron in extracellular space may induce oxidative stress.

Moreover, both the 4- and the 6-week ZnD diets lowered serum zinc and increased serum iron concentrations. Data concerning the relationship between depression and serum iron concentration are inconsistent (Maes et al. 1996; Rybka et al. 2013). In contrast, decreased serum zinc level associated with depression has been repeatedly observed (Siwek et al. 2013b) and has been demonstrated through a meta-analysis of clinical studies (Swardfager et al. 2013). The altered levels of magnesium and calcium in serum following zinc depletion suggest redistribution of these elements after dietary zinc regimen, although peripheral alterations were in the present study not accompanied by central alterations. In this study, the 4-week ZnD diet increased serum magnesium concentration. In animal models of depression based on stress exposure and in olfactory bulbectomy model, no differences in serum magnesium levels were demonstrated (Zieba et al. 2000); however, higher serum magnesium concentrations have been reported in depressed patients, compared to healthy controls, yet the data are not consistent (Serefko et al. 2013). Lowered level of magnesium in the Hp was recently shown in post-mortem study of suicide subjects (Sowa-Kucma et al. 2013).

Also, data regarding the relationship between serum copper concentration and depression are not consistent, but higher serum copper levels in patients suffering from depression have been reported (Manser et al. 1989; Schlegel-Zawadzka et al. 1999). In the present study, we did not observe differences in copper levels either in serum or brain tissues. Likewise, the study of zinc deficiency in mice, which demonstrated depression-like behavior and decreased serum zinc level, did not show differences in serum copper levels (Mlyniec et al. 2014b).

Associations between the increase in the lipid (TBARS) and protein (PCC) oxidation, pro-inflammatory cytokine (IL-1α and β) levels, decrease of zinc, and increase of iron levels have been noted previously in in vivo (Arruda et al. 2013; Mehrpouya et al. 2015) and in vitro/cell culture (Tate et al. 1999; Wessels et al. 2013) experiments. Moreover, a link of depression-like behavior with the enhancement of oxidative stress/pro-inflammatory status and with iron content in other experimental models and in clinical studies (Wayhs et al. 2010; Lopresti et al. 2014; Spanemberg et al. 2014; Mehrpouya et al. 2015; Tsai and Huang. 2015) was demonstrated. Increased oxidative stress as well as pro-inflammatory cytokines may lead to NMDAR activation (Leonard and Maes. 2012; Maes et al. 2012a; Felger and Lotrich 2013; Myint and Kim 2014). Furthermore, a mixture of pro-inflammatory cytokines, containing IL-1β, was shown to increase glutamate release (Ida et al. 2008), whereas administration of IL-1β induced depression-like behavior (Maes et al. 2012a). Thus, increased levels of pro-inflammatory cytokines and enhancement of oxidation in the Hp and PFC of the ZnD rats may contribute to increased release of glutamate, NMDAR activation, and depression-like behavior.

## Conclusions

Dietary zinc restriction induces peripheral and central alterations of bio-elements (namely zinc and iron) and enhances oxidative damage and pro-inflammatory status. These alterations share some similarities to those observed after exposure to stress and in depression, which further highlights that zinc deficiency [recently proposed as a model of depression (Whittle et al. 2009; Mlyniec et al. 2013; Doboszewska et al. 2015)] and depression may share a common pathophysiology. The changes observed in the present study may have a link to depression-like behavior in the zinc deficiency paradigm and may lead to/result from enhanced glutamate transmission through NMDAR.

## References

[CR1] Arruda LF, Arruda SF, Campos NA, de Valencia FF, Siqueira EM (2013). Dietary iron concentration may influence aging process by altering oxidative stress in tissues of adult rats. PLoS One.

[CR2] Bilgic B, Pfefferbaum A, Rohlfing T, Sullivan EV, Adalsteinsson E (2012). MRI estimates of brain iron concentration in normal aging using quantitative susceptibility mapping. Neuroimage.

[CR3] Bonaventura P, Benedetti G, Albarede F, Miossec P (2015). Zinc and its role in immunity and inflammation. Autoimmun Rev.

[CR4] Cheah JH, Kim SF, Hester LD, Clancy KW, Patterson SE, Papadopoulos V, Snyder SH (2006). NMDA receptor-nitric oxide transmission mediates neuronal iron homeostasis via the GTPase Dexras1. Neuron.

[CR5] Chu Y, Mouat MF, Harris RB, Coffield JA, Grider A (2003). Water maze performance and changes in serum corticosterone levels in zinc-deprived and pair-fed rats. Physiol Behav.

[CR79] Cole TB, Wenzel HJ, Kafer KE, Schwartzkroin PA, Palmiter RD (1999) Elimination of zinc from synaptic vesicles in the intact mouse brain by disruption of the ZnT3 gene. Proc Natl Acad Sci 96:1716–1721 10.1073/pnas.96.4.1716 PMC15571 9990090

[CR6] Dinarello CA, Simon A, van der Meer JW (2012). Treating inflammation by blocking interleukin-1 in a broad spectrum of diseases. Nat Rev Drug Discov.

[CR7] Doboszewska U, Sowa-Kucma M, Mlyniec K, Pochwat B, Holuj M, Ostachowicz B, Pilc A, Nowak G, Szewczyk B (2015). Zinc deficiency in rats is associated with up-regulation of hippocampal NMDA receptor. Prog Neuropsychopharmacol Biol Psychiatry.

[CR8] Dou X, Tian X, Zheng Y, Huang J, Shen Z, Li H, Wang X, Mo F, Wang W, Wang S, Shen H (2014). Psychological stress induced hippocampus zinc dyshomeostasis and depression-like behavior in rats. Behav Brain Res.

[CR9] Felger JC, Lotrich FE (2013). Inflammatory cytokines in depression: neurobiological mechanisms and therapeutic implications. Neuroscience.

[CR10] Ghasemi M, Phillips C, Trillo L, De Miguel Z, Das D, Salehi A (2014). The role of NMDA receptors in the pathophysiology and treatment of mood disorders. Neurosci Biobehav Rev.

[CR11] Hagmeyer S, Haderspeck JC, Grabrucker AM (2015). Behavioral impairments in animal models for zinc deficiency. Front Behav Neurosci.

[CR12] Ida T, Hara M, Nakamura Y, Kozaki S, Tsunoda S, Ihara H (2008). Cytokine-induced enhancement of calcium-dependent glutamate release from astrocytes mediated by nitric oxide. Neurosci Lett.

[CR13] Jurowski K, Szewczyk B, Nowak G, Piekoszewski W (2014). Biological consequences of zinc deficiency in the pathomechanisms of selected diseases. J Biol Inorg Chem.

[CR14] Kim J, Wessling-Resnick M (2014). Iron and mechanisms of emotional behavior. J Nutr Biochem.

[CR15] Kopanski Z, Schlegel-Zawadzka M, Piekoszewski W, Sadlik K, Sibiga W (2000). The disturbances of magnesium in patients with thromboembolic complications after a cholecystectomy. Thromb Res.

[CR16] Leonard B, Maes M (2012). Mechanistic explanations how cell-mediated immune activation, inflammation and oxidative and nitrosative stress pathways and their sequels and concomitants play a role in the pathophysiology of unipolar depression. Neurosci Biobehav Rev.

[CR17] Li Y, Zheng Y, Qian J, Chen X, Shen Z, Tao L, Li H, Qin H, Li M, Shen H (2012). Preventive effects of zinc against psychological stress-induced iron dyshomeostasis, erythropoiesis inhibition, and oxidative stress status in rats. Biol Trace Elem Res.

[CR18] Lopresti AL, Maker GL, Hood SD, Drummond PD (2014). A review of peripheral biomarkers in major depression: the potential of inflammatory and oxidative stress biomarkers. Prog Neuropsychopharmacol Biol Psychiatry.

[CR19] Maes M, Van de Vyvere J, Vandoolaeghe E, Bril T, Demedts P, Wauters A, Neels H (1996). Alterations in iron metabolism and the erythron in major depression: further evidence for a chronic inflammatory process. J Affect Disord.

[CR20] Maes M, Fisar Z, Medina M, Scapagnini G, Nowak G, Berk M (2012). New drug targets in depression: inflammatory, cell-mediated immune, oxidative and nitrosative stress, mitochondrial, antioxidant, and neuroprogressive pathways. And new drug candidates–Nrf2 activators and GSK-3 inhibitors. Inflammopharmacology.

[CR21] Maes M, Fisar Z, Medina M, Scapagnini G, Nowak G, Berk M (2012). New drug targets in depression: inflammatory, cell-mediated immune, oxidative and nitrosative stress, mitochondrial, antioxidant, and neuroprogressive pathways. And new drug candidates–Nrf2 activators and GSK-3 inhibitors. Inflammopharmacology.

[CR22] Manser WW, Khan MA, Hasan KZ (1989). Trace element studies on Karachi population. Part IV: blood copper, zinc, magnesium and lead levels in psychiatric patients with depression, mental retardation and seizure disorders. J Pak Med Assoc.

[CR23] Marsden WN (2011). Stressor-induced NMDAR dysfunction as a unifying hypothesis for the aetiology, pathogenesis and comorbidity of clinical depression. Med Hypotheses.

[CR24] Mayer ML, Westbrook GL (1987). Permeation and block of N-methyl-d-aspartic acid receptor channels by divalent cations in mouse cultured central neurones. J Physiol.

[CR25] Mehrpouya S, Nahavandi A, Khojasteh F, Soleimani M, Ahmadi M, Barati M (2015). Iron administration prevents BDNF decrease and depressive-like behavior following chronic stress. Brain Res.

[CR26] Mlyniec K, Davies CL, Budziszewska B, Opoka W, Reczynski W, Sowa-Kucma M, Doboszewska U, Pilc A, Nowak G (2012). Time course of zinc deprivation-induced alterations of mice behavior in the forced swim test. Pharmacol Rep.

[CR27] Mlyniec K, Budziszewska B, Reczynski W, Sowa-Kucma M, Nowak G (2013). The role of the GPR39 receptor in zinc deficient-animal model of depression. Behav Brain Res.

[CR28] Mlyniec K, Davies CL, de Aguero Sanchez IG, Pytka K, Budziszewska B, Nowak G (2014). Essential elements in depression and anxiety. Part I. Pharmacol Rep.

[CR29] Mlyniec K, Ostachowicz B, Krakowska A, Reczynski W, Opoka W, Nowak G (2014). Chronic but not acute antidepresant treatment alters serum zinc/copper ratio under pathological/zinc-deficient conditions in mice. J Physiol Pharmacol.

[CR30] Mlyniec K, Gawel M, Doboszewska U, Starowicz G, Pytka K, Davies CL, Budziszewska B (2015). Essential elements in depression and anxiety. Part II. Pharmacol Rep.

[CR31] Moylan S, Berk M, Dean OM, Samuni Y, Williams LJ, O’Neil A, Hayley AC, Pasco JA, Anderson G, Jacka FN, Maes M (2014). Oxidative and nitrosative stress in depression: why so much stress?. Neurosci Biobehav Rev.

[CR32] Myint AM, Kim YK (2014). Network beyond IDO in psychiatric disorders: revisiting neurodegeneration hypothesis. Prog Neuropsychopharmacol Biol Psychiatry.

[CR33] Nakamichi N, Ohno H, Nakamura Y, Hirai T, Kuramoto N, Yoneda Y (2002). Blockade by ferrous iron of Ca^2+^ influx through N-methyl-D-aspartate receptor channels in immature cultured rat cortical neurons. J Neurochem.

[CR34] Opoka W, Sowa-Kucma M, Stachowicz K, Ostachowicz B, Szlosarczyk M, Stypula A, Mlyniec K, Maslanka A, Bas B, Lankosz M, Nowak G (2010). Early lifetime zinc supplementation protects zinc-deficient diet-induced alterations. Pharmacol Rep.

[CR35] Oteiza PI (2012). Zinc and the modulation of redox homeostasis. Free Radic Biol Med.

[CR36] Paoletti P, Vergnano AM, Barbour B, Casado M (2009). Zinc at glutamatergic synapses. Neuroscience.

[CR37] Paxinos G, Watson C (1998). The rat brain in stereotaxic coordinates.

[CR38] Prasad AS (2012). Discovery of human zinc deficiency: 50 years later. J Trace Elem Med Biol.

[CR39] Prasad AS (2014). Zinc is an antioxidant and anti-inflammatory agent: its role in human health. Front Nutr.

[CR40] Ramos P, Santos A, Pinto NR, Mendes R, Magalhaes T, Almeida A (2014). Iron levels in the human brain: a post-mortem study of anatomical region differences and age-related changes. J Trace Elem Med Biol.

[CR41] Rybka J, Kedziora-Kornatowska K, Banas-Lezanska P, Majsterek I, Carvalho LA, Cattaneo A, Anacker C, Kedziora J (2013). Interplay between the pro-oxidant and antioxidant systems and proinflammatory cytokine levels, in relation to iron metabolism and the erythron in depression. Free Radic Biol Med.

[CR42] Sanacora G, Treccani G, Popoli M (2012). Towards a glutamate hypothesis of depression: an emerging frontier of neuropsychopharmacology for mood disorders. Neuropharmacology.

[CR43] Schipper HM (2012). Neurodegeneration with brain iron accumulation—clinical syndromes and neuroimaging. Biochim Biophys Acta.

[CR44] Schlegel-Zawadzka M, Zieba A, Dudek D, Zak-Knapik J, Nowak G (1999). Is serum copper a “trait marker” of unipolar depression? A preliminary clinical study. Pol J Pharmacol.

[CR45] Serefko A, Szopa A, Wlaz P, Nowak G, Radziwon-Zaleska M, Skalski M, Poleszak E (2013). Magnesium in depression. Pharmacol Rep.

[CR46] Siwek M, Sowa-Kucma M, Dudek D, Styczen K, Szewczyk B, Kotarska K, Misztakk P, Pilc A, Wolak M, Nowak G (2013). Oxidative stress markers in affective disorders. Pharmacol Rep.

[CR47] Siwek M, Szewczyk B, Dudek D, Styczen K, Sowa-Kucma M, Mlyniec K, Siwek A, Witkowski L, Pochwat B, Nowak G (2013). Zinc as a marker of affective disorders. Pharmacol Rep.

[CR48] Smaga I, Krzyżanowska W, Pomierny B, Budziszewska B, Pilc A, Nowak G (2014) The role of neurotoxicity in depression. In: Kostrzewa RM (eds) Handbook of neurotoxicity. Springer-Verlag, New York, pp 1567–1593

[CR49] Sowa-Kucma M, Szewczyk B, Sadlik K, Piekoszewski W, Trela F, Opoka W, Poleszak E, Pilc A, Nowak G (2013). Zinc, magnesium and NMDA receptor alterations in the hippocampus of suicide victims. J Affect Disord.

[CR50] Spanemberg L, Caldieraro MA, Vares EA, Wollenhaupt-Aguiar B, Kauer-Sant’Anna M, Kawamoto SY, Galvao E, Parker G, Fleck MP (2014). Biological differences between melancholic and nonmelancholic depression subtyped by the CORE measure. Neuropsychiatr Dis Treat.

[CR51] Steffens DC, Tupler LA, Ranga K, Krishnan R (1998). Magnetic resonance imaging signal hypointensity and iron content of putamen nuclei in elderly depressed patients. Psychiatry Res.

[CR52] Stys PK, You H, Zamponi GW (2012). Copper-dependent regulation of NMDA receptors by cellular prion protein: implications for neurodegenerative disorders. J Physiol.

[CR53] Summersgill H, England H, Lopez-Castejon G, Lawrence CB, Luheshi NM, Pahle J, Mendes P, Brough D (2014). Zinc depletion regulates the processing and secretion of IL-1beta. Cell Death Dis.

[CR54] Swardfager W, Herrmann N, Mazereeuw G, Goldberger K, Harimoto T, Lanctot KL (2013). Zinc in depression: a meta-analysis. Biol Psychiatry.

[CR55] Szewczyk B (2013). Zinc homeostasis and neurodegenerative disorders. Front Aging Neurosci.

[CR56] Szewczyk B, Kubera M, Nowak G (2011). The role of zinc in neurodegenerative inflammatory pathways in depression. Prog Neuropsychopharmacol Biol Psychiatry.

[CR57] Szewczyk B, Palucha-Poniewiera A, Poleszak E, Pilc A, Nowak G (2012). Investigational NMDA receptor modulators for depression. Expert Opin Investig Drugs.

[CR58] Takeda A, Minami A, Takefuta S, Tochigi M, Oku N (2001). Zinc homeostasis in the brain of adult rats fed zinc-deficient diet. J Neurosci Res.

[CR59] Takeda A, Hirate M, Tamano H, Oku N (2003). Release of glutamate and GABA in the hippocampus under zinc deficiency. J Neurosci Res.

[CR60] Takeda A, Tamano H, Tochigi M, Oku N (2005). Zinc homeostasis in the hippocampus of zinc-deficient young adult rats. Neurochem Int.

[CR61] Takeda A, Tamano H, Kan F, Itoh H, Oku N (2007). Anxiety-like behavior of young rats after 2-week zinc deprivation. Behav Brain Res.

[CR62] Takeda A, Tamano H, Kan F, Hanajima T, Yamada K, Oku N (2008). Enhancement of social isolation-induced aggressive behavior of young mice by zinc deficiency. Life Sci.

[CR63] Takeda A, Yamada K, Tamano H, Fuke S, Kawamura M, Oku N (2008). Hippocampal calcium dyshomeostasis and long-term potentiation in 2-week zinc deficiency. Neurochem Int.

[CR64] Takeda A, Tamano H, Ogawa T, Takada S, Ando M, Oku N, Watanabe M (2012). Significance of serum glucocorticoid and chelatable zinc in depression and cognition in zinc deficiency. Behav Brain Res.

[CR65] Tao L, Zheng Y, Shen Z, Li Y, Tian X, Dou X, Qian J, Shen H (2013). Psychological stress-induced lower serum zinc and zinc redistribution in rats. Biol Trace Elem Res.

[CR66] Tate DJ, Miceli MV, Newsome DA (1999). Zinc protects against oxidative damage in cultured human retinal pigment epithelial cells. Free Radic Biol Med.

[CR67] Tsai MC, Huang TL (2015). Thiobarbituric acid reactive substances (TBARS) is a state biomarker of oxidative stress in bipolar patients in a manic phase. J Affect Disord.

[CR68] Vashum KP, McEvoy M, Milton AH, McElduff P, Hure A, Byles J, Attia J (2014). Dietary zinc is associated with a lower incidence of depression: findings from two Australian cohorts. J Affect Disord.

[CR69] Vlachova V, Zemkova H, Vyklicky L (1996). Copper modulation of NMDA responses in mouse and rat cultured hippocampal neurons. Eur J Neurosci.

[CR70] Wang L, Wang W, Zhao M, Ma L, Li M (2008). Psychological stress induces dysregulation of iron metabolism in rat brain. Neuroscience.

[CR81] Ward RJ, Zucca FA, Duyn JH, Crichton RR, Zecca L (2014). The role of iron in brain ageing and neurodegenerative disorders. Lancet Neurol.

[CR71] Watanabe M, Inoue Y, Sakimura K, Mishina M (1992). Developmental changes in distribution of NMDA receptor channel subunit mRNAs. NeuroReport.

[CR72] Wayhs CA, Manfredini V, Sitta A, Deon M, Ribas G, Vanzin C, Biancini G, Ferri M, Nin M, Barros HM, Vargas CR (2010). Protein and lipid oxidative damage in streptozotocin-induced diabetic rats submitted to forced swimming test: the insulin and clonazepam effect. Metab Brain Dis.

[CR73] Wessels I, Haase H, Engelhardt G, Rink L, Uciechowski P (2013). Zinc deficiency induces production of the proinflammatory cytokines IL-1beta and TNFalpha in promyeloid cells via epigenetic and redox-dependent mechanisms. J Nutr Biochem.

[CR83] Whitfield DR, Vallortigara J, Alghamdi A, Hortobagyi T, Ballard C, Thomas AJ, O'Brien JT, Aarsland D, Francis PT (2015). Depression and synaptic zinc regulation in Alzheimer disease, dementia with lewy bodies, and Parkinson disease dementia. Am J Geriatr Psychiatry.

[CR74] Whittle N, Lubec G, Singewald N (2009). Zinc deficiency induces enhanced depression-like behaviour and altered limbic activation reversed by antidepressant treatment in mice. Amino Acids.

[CR85] Wu LJ, Leenders AG, Cooperman S, Meyron-Holtz E, Smith S, Land W, Tsai RY, Berger UV, Sheng ZH, Rouault TA (2004). Expression of the iron transporter ferroportin in synaptic vesicles and the blood-brain barrier. Brain Res.

[CR75] Yu S, Feng Y, Shen Z, Li M (2011). Diet supplementation with iron augments brain oxidative stress status in a rat model of psychological stress. Nutrition.

[CR87] Zhang HY, Song N, Jiang H, Bi MX, Xie JX (2014). Brain-derived neurotrophic factor and glial cell line-derived neurotrophic factor inhibit ferrous iron influx via divalent metal transporter 1 and iron regulatory protein 1 regulation in ventral mesencephalic neurons. Biochim Biophys Acta.

[CR76] Zheng W, Monnot AD (2012). Regulation of brain iron and copper homeostasis by brain barrier systems: implication in neurodegenerative diseases. Pharmacol Ther.

[CR77] Zieba A, Kata R, Dudek D, Schlegel-Zawadzka M, Nowak G (2000). Serum trace elements in animal models and human depression: part III. Magnesium. Relationship with copper. Hum Psychopharmacol.

